# Yeast-based assay identifies novel Shh/Gli target genes in vertebrate development

**DOI:** 10.1186/1471-2164-13-2

**Published:** 2012-01-03

**Authors:** Luis A Milla, Claudio R Cortés, Christian Hodar Q, Maritza G Oñate, Veronica Cambiazo, Shawn M Burgess, Verónica Palma

**Affiliations:** 1 Faculty of Sciences, Universidad de Chile, Santiago, Chile; 2 Laboratorio de Bioinformática y Expresión Génica, INTA, Universidad de Chile, Santiago, Chile; 3 FONDAP Center for Genome Regulation, Facultad de Ciencias, Universidad de Chile; 4 National Human Genome Research Institute, National Institutes of Health, Bethesda, Maryland, USA

**Keywords:** Hh/Gli targets, zebrafish, purmorphamine, cyclopamine, neogenin 1, c-myc, sfrp2

## Abstract

**Background:**

The increasing number of developmental events and molecular mechanisms associated with the Hedgehog (Hh) pathway from *Drosophila *to vertebrates, suggest that gene regulation is crucial for diverse cellular responses, including target genes not yet described. Although several high-throughput, genome-wide approaches have yielded information at the genomic, transcriptional and proteomic levels, the specificity of Gli binding sites related to direct target gene activation still remain elusive. This study aims to identify novel putative targets of Gli transcription factors through a protein-DNA binding assay using yeast, and validating a subset of targets both *in-vitro *and *in-vivo*. Testing in different Hh/Gli gain- and loss-of-function scenarios we here identified known (e.g., *ptc1*) and novel Hh-regulated genes in zebrafish embryos.

**Results:**

The combined yeast-based screening and MEME/MAST analysis were able to predict Gli transcription factor binding sites, and position mapping of these sequences upstream or in the first intron of promoters served to identify new putative target genes of Gli regulation. These candidates were validated by qPCR in combination with either the pharmacological Hh/Gli antagonist cyc or the agonist pur in Hh-responsive C3H10T1/2 cells. We also used small-hairpin RNAs against Gli proteins to evaluate targets and confirm specific Gli regulation their expression. Taking advantage of mutants that have been identified affecting different components of the Hh/Gli signaling system in the zebrafish model, we further analyzed specific novel candidates. Studying Hh function with pharmacological inhibition or activation complemented these genetic loss-of-function approaches. We provide evidence that in zebrafish embryos, Hh signaling regulates *sfrp2, neo1*, and *c-myc *expression *in-vivo*.

**Conclusion:**

A recently described yeast-based screening allowed us to identify new Hh/Gli target genes, functionally important in different contexts of vertebrate embryonic development.

## Background

The Sonic hedgehog (Shh)/Gli pathway orchestrates several processes such as cell proliferation, differentiation, and stem cell maintenance [1]. Shh acts as a secreted protein ligand that binds to the 12-transmembrane receptor Ptc1 (Patched/Ptch/Ptch1). This binding releases Ptc1 repression of the 7-transmembrane co-receptor Smoothened, which in turn favours intracellular accumulation of activator forms of the Gli transcription factors (Gli-A). In vertebrates, at least three Gli proteins (Gli1, Gli2 and Gli3) have been described. These zinc-finger transcription factors enter the nucleus and bind specific sequences (GACCACCCA) in the genome known as Gli Binding sites (GBS), promoting expression of target genes, including *ptc1 *and *gli1 *themselves [2]. In the absence of ligand, Ptc1 represses Smoothened activity and the repressor forms of the transcription factors Gli (Gli-R) enter the nucleus and inhibit Hh target gene transcription. The relative ratio of Gli-A/Gli-R forms is considered to be crucial for interpreting the extracellular Hh gradient and for determining concentration-dependent cell fates [3,4]. Due the multiple developmental and growth processes where the Hh/Gli pathway has been implicated, it is likely that the cellular outcome involves a high number of target genes. To date, based on the identification of a Gli binding motif within their regulatory sequences, no more than a dozen target genes have been characterized to be activated or inhibited by Gli activity in vertebrates. Several *in silico *and experimental genomic analyses, such as ChIP-seq and ChIP-Chip, have been recently made to identify new gene direct targets, mainly identifying putative GBS near the transcriptional start site of genes [5-8]. However, it has been shown that different genomic strategies yield different hedgehog target lists.

In order to contribute to the identification of new GBS we applied a recently described yeast-based screen [9] using mouse and zebrafish genomic libraries. This versatile yeast strategy allowed us to rapidly and efficiently identify genomic targets of Gli-binding proteins. Bioinformatic analyses (MEME/MAST) were performed to determine the presence of enriched elements upstream or in the first intron of putative targets identified by our one-hybrid assay. Using this heterologous approach, we were able to identify several novel Gli-binding sequences located close to genes previously not connected to the Shh/Gli pathway. Due to the high conservation of the Hh/Gli pathway in vertebrates, some of the identified putative targets were further analyzed, both *in-vitro *using a mouse Hh reporter fibroblast cell line, and *in-vivo *in zebrafish embryos, confirming their predicted regulation through Hh loss and gain-of-function experiments. The high rate of identified target genes supports our unbiased approach and shows the potency of this method for finding novel target genes.

## Results

### Yeast-based screens identify Gli protein-binding sequences

In order to identify possible GBS, we used our recently described yeast-based assay that uses mouse and zebrafish libraries with random genomic fragments upstream of the yeast URA3 promoter [9]. The zebrafish library had an average genomic fragment size of 300 bp, and contains approximately 3 × 10^7 ^independent clones providing a 4- to 6-fold coverage of the genome, while the mouse library contains 1.7 × 10^7 ^independent clones with an average size of 700 bp, representing an average of 3- to 4-fold coverage of the genome. *MATa *yeast containing the genome library fragment plasmids were mated separately to *MATα *yeast containing an expression plasmid for either the full-length zebrafish Gli1 or the zinc-finger domain of the mouse Gli2 protein, following a standard two-hybrid mating protocol, screening afterwards a total of 611 clones [10]. Clones were then re-screened for a URA3 phenotype and the genomic fragment was PCR amplified using primers flanking the genomic library. Sequencing a total of 512 single product colony-PCR reactions, we successfully obtained 235 potential binding sequences for the mouse library, and 197 for the zebrafish library.

### MEME and MAST analysis predict multiple Shh/Gli target genes

The consensus GBS, GACCACCCA, was first described by the Vogelstein group [11]. Several other genes that have been reported as targets of Gli1 in different biological processes, posses motifs with distinct degrees of similarity to the consensus sequences in their upstream regions. These motifs were able to bind a recombinant form of Gli1 in gel-shift assays [12]. From the 432 sequences obtained from the yeast assay, we manually curated them based on two criteria: first, we considered those sequences mapping within 20 kb of the transcriptional start of a gene or to the first intron, and second, we ruled out sequences that have been described as repetitive in multiple genome assemblies. We therefore did not consider the intergenic repetitive sequences, although they show tandem repeats that probably could bind the Gli transcription factors. Nevertheless, for the purpose of this study, they were discarded based on the fact that they do not show the Gli consensus binding sites or other known transcription factor binding sites. Next, we selected for further analysis and logo generation the mouse sequences since they fulfilled our request of mapping criteria. This yielded a list of 128, mouse sequences, with final 66 non-redundant sequences (Additional File 1), mapped to specific genes based on shortest distance to the transcriptional start site. These sequences were used as input for a MEME search.

**Figure 1:**
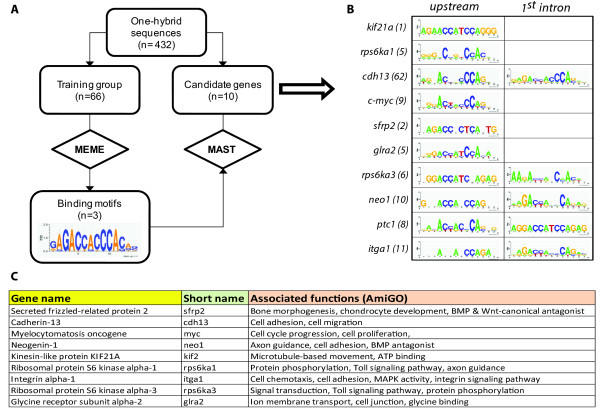
**Motif search strategy**. A) Diagram of the bioinformatic workflow for sequence analysis. A subgroup of sequences from the one-hybrid assay was used to search for motifs using MEME. Logos on the right represent the position-dependent matrix based in the alignment for GLI transcription factor candidate motifs. The matrix was also used as input to the MAST algorithm to identify potential Gli-binding sites in non-coding regions of candidate genes. B) Motifs identified with MEME/MAST strategy. Logos represent the alignment of the potential binding sites identified in each sequence in both upstream (left column) and intronic (right column) for nine candidate genes. Numbers of potential binding sites are indicated in brackets next to gene name. C) Gene ontology table for selected putative target genes found in (B).

As shown in Figure 1A, we used the MEME [13,14] algorithm to identify overrepresented motifs in the group of 128 sequences (Additional File 1). A predicted site, a 14 bp motif (Figure 1A), contained the core consensus of GBS as described in [11]. The position-dependent scoring matrix for the 14 bp motif was used as input to the MAST algorithm to search similar motifs in a 20 kb upstream region and in the first intron of 10 candidate genes, including *ptc1*, candidate genes were selected based on gene ontology associations, to cover a wide array of cellular and developmental processes (Figure 1C). Figure 1B shows the representative logos of potential Gli-binding sites identified in the non-coding regions of the selected genes, for each gene the number of sequences that matched to the 14 bp motif is indicated in the brackets right next to gene name.

We also checked the sequences for interspecies conservation, finding a high percentage for some of them in a murine/human comparison (like *neo1 *and *cdh13*), partial human/murine conservation for others (*kif21a, rps6ka3*), some with murine-only conservation (like *itga1*), and finally some with no conservation at all (*sfrp2*) (Additional File 2).

### Putative Gli-targets genes display altered mRNA levels after pharmacological blockage or activation of Hh/Gli signaling pathway

Assessing whether regulatory elements are occupied *in-vivo *requires experimental confirmation. In order to address whether any of the putative 9 target genes are affected by Hh pathway loss of function, we made use of the reporter cell line C3H10T1/2 and assayed the candidate target genes for their response to Hh signaling by quantitative RT-PCR (qPCR). Cells were cultured in the presence of the Hh/Gli pathway inhibitor cyclopamine (cyc) (10 μM) and three time points were considered (6, 12 and 24 hours) to account for differences in the temporal transcriptional response of putative Gli-target genes and for differences in the stability of mRNAs produced under Gli control. Vehicle control (EtOH) was used as reference.

**Figure 2:**
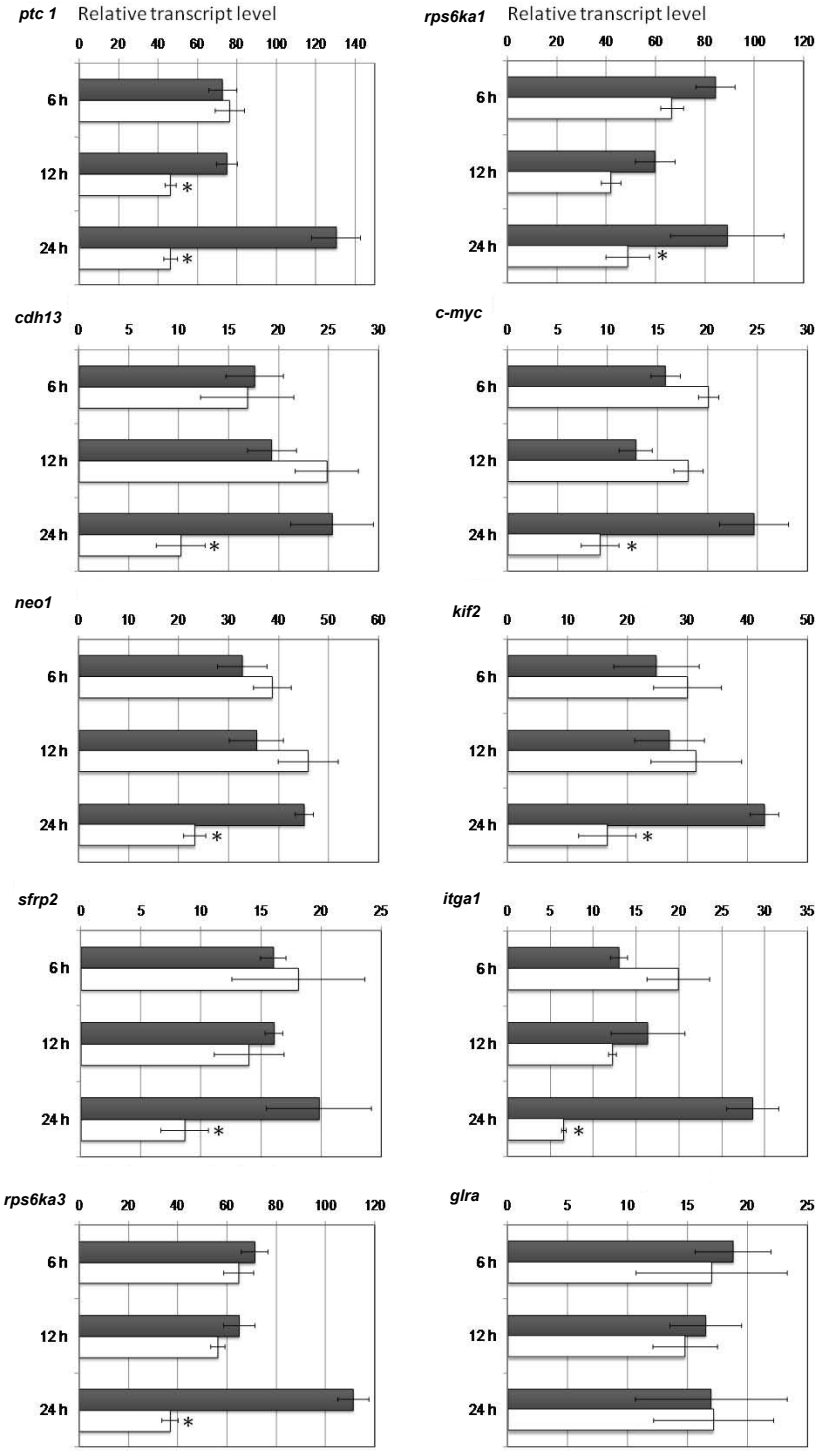
**Expression analysis of Gli-target candidate genes in control and cyc treated-cells**. Real-time RT-PCR analysis of expression of genes control gene *ptc1 *(*Patched1*), *cdh13 *(*cadherin 13*), *neo1 *(*Neogenin 1*), *sfrp2 *(*Secreted Frizzled Related Protein 2*), *rps6ka3 *(*ribosomal protein S6 kinase polypeptide 3*), *rps6ka1 *(*ribosomal protein S6 kinase polypeptide 1*), *c-myc *(*myelocytomatosis oncogene*), *kif2 *(*Kinesin Heavy Chain Member 2*), *itga1 *(*integrin alpha 1*) and *glra2 *(*glycine receptor, alpha 2 subunit*) in control cells and in cells treated with 10 μM cyc or control (vehicle EtOH) for 6, 12 or 24 hours before harvesting. Data shown are transcript levels relative to housekeeping gene *gapdh *(glyceraldehyde 3-phospate dehydrogenase). Values correspond to the mean and SE of at least triplicate determinations and asterisks indicate significant difference between control and treated cells (ANOVA, *p *< 0.05). Black bars, control samples and white bars, inhibitor treated samples.

**Figure 3:**
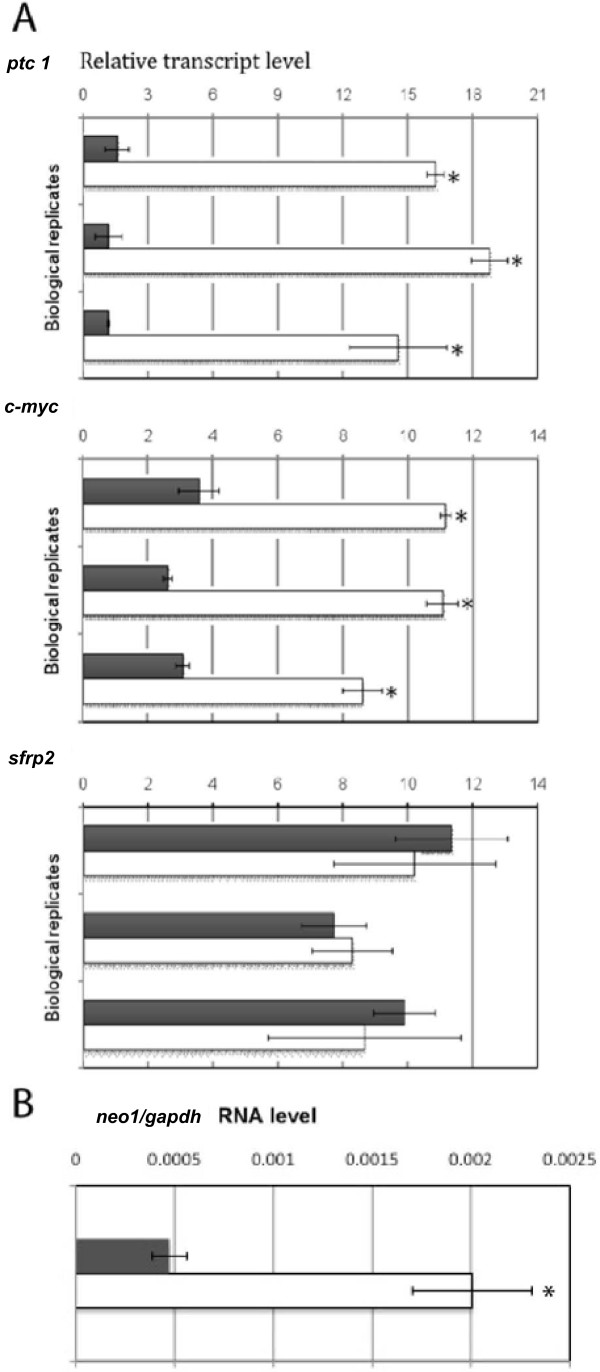
**Pharmacological gain of function of the Shh/Gli pathway activates transcription of novel target genes**. A) C3H10T1/2 cells were cultured for 48 h in 10 μM pur or its control (vehicle DMSO) and processed for qPCR. *c-myc *shows significant differences in mRNA levels, whereas *sfrp2 *does not. *ptc 1 *is shown as a positive control. B) *neo1 *mRNA levels are increased after the same 48 h treatment of C3H10T1/2 cells, graph showed separately due to use of TaqMan probes.

All the selected candidate genes showed reductions in their mRNA levels after 24-hour cyc treatments except *glra *(Figure 2). To corroborate Hh regulation we performed gain of function experiments using purmorphamine (pur), a proven Smoothened agonist [15] for a subset of candidates, namely *neo1, c-myc *and *sfrp2 *based on their implication in developmental processes. Cells were treated for 24 hours and 48 hours before RNA extraction and qPCR processing. *ptc1*, a well known Hh transcriptional readout was used as positive control. For both *c-myc *and *neo1 *significant changes could be observed after 24-hours of treatment, nevertheless the increases was less pronounced in comparison to *ptc1*. In order to proof that the effects of Hh signaling were direct we included experiments with cycloheximide (CHX), obtaining similar results (Additional File 3). Increase in transcript levels of the selected target genes became clearly evident after 48 hours of treatment. Sfrp2 did not show any changes in expression for either time point (Figure 3). *sfrp2 *invariant mRNA levels possibly points to a context dependent case of regulation. Expression changes were calculated related to vehicle treatments (DMSO).

Based on the Gli-specific effect on transcription of novel target genes obtained by the screening and further *in-vitro *validation experiments for *neo1, c-myc *and *sfrp2*, we also verified Hh regulation of these genes by whole mount *in situ hybridization *(ISH) in zebrafish embryos. Besides allowing a relatively rapid verification of gene expression in different Hh-manipulated embryos, this approach provides spatial information and insights about the nature of regulation by Hh signaling. Because the Shh/Gli pathway plays a central role in neural development, particularly in the development of the central nervous system (CNS), we choose these known developmental genes not previously reported to be regulated by Shh signaling.

### *Neogenin 1 *is positively regulated by the Shh/Gli pathway in in the CNS

**Figure 4:**
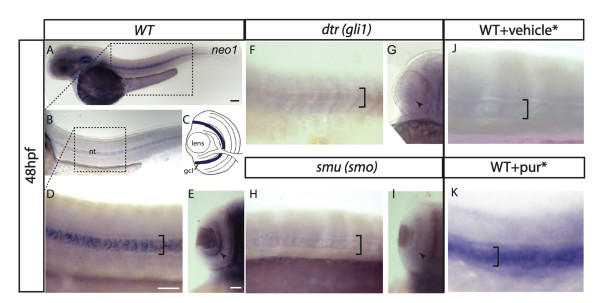
***neo1 *expression is reduced in Shh/Gli pathway mutants and increased on Shh/Gli pharmacological activation**. *neo1 *is expressed broadly in the developing CNS (A). Expression in 48 hpf embryo can be detected in the otic vesicle, in the notochord (B, close-up D, black bracket) and in the eye (E, black arrowhead), probably in the retinal ganglion cell layer (gcl). (C) Cartoon of a 48 hpf zebrafish eye. Blue line shows *neo1 *expression in the gcl. At 48 hpf *neo1 *expression is diminished in gli1 (*dtr*) and smu (*smo*) mutants, specifically in the notochord (F, H, bracket) and the gcl in the optic placode (G, I; black arrowhead) in comparison to WT (D, E). (J, K) *neo1 *mRNA is increased in notochord. Note that (J) and (K) controls were developed at different times in comparison to (D) in order to match to their corresponding pur treatment (K). Anterior is towards left, lateral views are showed except in (E, G, I, K) with dorsal views anterior up. Scale bars; 50 μm except in A (100 μm). WT; wild type. gcl; retinal-ganglion cell layer. DMSO is the vehicle for pur.

Neogenin 1 is a multifunctional transmembrane receptor belonging to the immunoglobulin superfamily with known roles in angiogenesis, progenitor proliferation and axon guidance [16]. At 48 hours post fertilization (hpf) *neo1 *expression was strongly detected in the wild- type CNS, where it was associated with proliferative zones (optic tectum, eye and diencephalon, Figure 4A-E). Of note, *neo1 *was also detected in the notochord (Figure 4D, bracket). ISH analysis shows no staining in the notochord and the retinal ganglion cell layer of the eye in *dtr *(gli1) and *smu *(smo) mutants (Figure 4F-I, bracket and arrowhead).

In addition to genetic loss-of-function, we complemented our analysis by treating embryos with the Shh/Gli activator pur. Of note, in particular notochord expression is increased when compared to mock DMSO treated embryos (Figure 4J, K, bracket).

### *c-myc *is regulated by the Shh/Gli pathway in the CNS and liver

**Figure 5:**
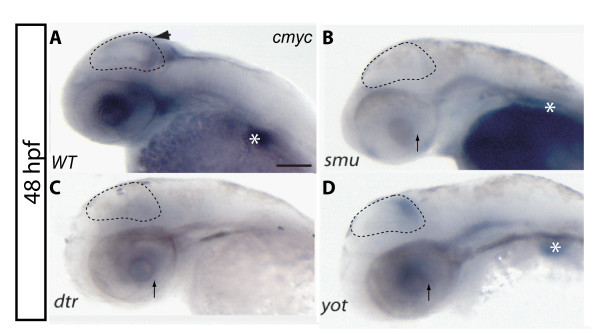
***C-myc *expression is lost in the CNS in Shh/Gli pathway mutants**. A) *c-myc *is normally expressed in the optic tectum (black arrowhead) and the developing eye. Strong expression is also detected in the liver (white asterisk). B) *smu *mutant embryos show no expression of *c-myc *in the CNS. C) *dtr *embryos have no *c-myc *in the eye (black arrow), tectum and liver. D) *yot *embryos still show labelling for *c-myc*, although reduced; in both the eye and optic tectum labelling is preserved. Liver also retains some *c-myc *labelling. All lateral views are dorsal up, anterior left.

We next asked whether *c-myc *(*myca*), a multifunctional gene directly related to cell cycle progression (G1/S phases) and implicated in a variety of cancers, is regulated by Shh [17]. At 48 hpf wild-type *c-myc *expression is found in the posterior margin of the optic tectum, recently identified as an important proliferative zone in the dorsal brain of teleosts (Figure 5A, arrow) [18,19]. The mRNA can also be detected in the eye and liver (Figure 5A asterisk). In the *smu *mutant, expression in CNS and liver was completely diminished (Figure 5B). *dtr *mutants did not show any *c-myc *staining; expression is regionally lost in the dorsal midbrain, eye and liver (Figure 5C, arrow indicates eye) whereas the *yot *(gli2) mutant has a reduced expression in the eye while *c-myc *was still detectable in the tectum and liver (Figure 5D). Thus, ISH analysis corroborated the changes in *c-myc *expression levels that were previously determined by qPCR assays.

### *sfrp2 *gene is regulated by the Shh pathway in slow muscle and pharyngeal arches

**Figure 6:**
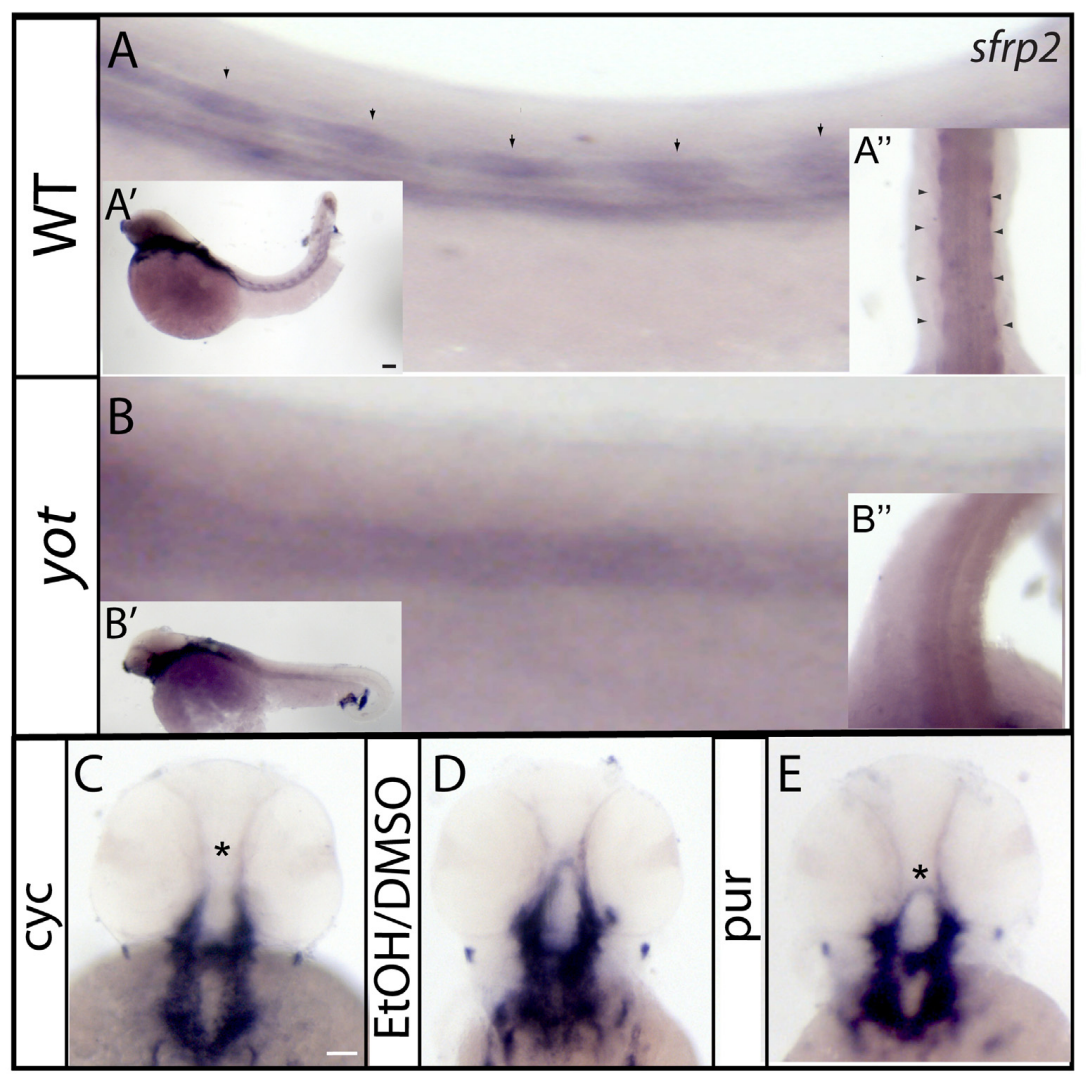
***sfrp2 *RNA levels are disturbed in Hh pathway mutants and after pharmacological treatments**. (A, A'') In WT embryos, *sfrp2 *expression is evident in adaxial cells. This expression is significantly down-regulated in *yot *embryos (B, B''). Pharmacological treatments with pur and cyc show altered expression in pharyngeal arches (asterisk in E, compare C, D and E for different levels of RNA). Anterior is towards left, lateral views are shown. Full lateral views of embryos are shown in A' and B'.

The *sfrp2 *gene encodes a protein that binds Wnt ligands through a cystein-rich domain (CRD). It has been implicated in both antagonism of Wnt signaling [20] and beta-catenin stabilization [21]. ISH analysis verified that *sfrp2 *transcripts can be detected in adaxial cells, pectoral fins and branchial arches by the 48 hpf stage, as previously reported [22]. Adaxial cell expression is absent in *yot *mutants, and expression is also regionally lost in the CNS (Figure 6B, B'''). Pectoral fins do not display any changes in *sfrp2 *expression. This is consistent with the reported alterations in myogenesis in *yot *mutants, where slow muscle genesis is affected [23]. While it is known that a gradient of Wnt signaling opposes the Shh signaling gradient in the neural tube, regulation of *sfrp2 *in the brain by Shh has not previously been demonstrated.

To further examine whether *sfrp2 *is regulated by Shh we used pharmacological gain and loss of function approaches. *Sfrp2 *expression is increased in pur treated embryos in comparison to its vehicle treatment (Figure 6E). Although cyc inhibitor does not ablate completely expression in the CNS, expression in the developing pharyngeal arch is abolished. (Figure 6C, see asterisk). This might be due to the fact that treatments were started at 8 hpf, due to an extremely high mortality rate in treatment initiated prior to that time. The failure to detect *sfrp2 *regulation in the first reporter cell assay demonstrates the necessity to test regulation in the proper, *in-vivo *context.

## Discussion

The Hedgehog pathway has been extensively studied. Many studies take advantage of high throughput approaches at different levels, for example by identifying the post-translational modifiers mediating Hh signaling [24], by mapping GBS and Gli responding genes *in silico *[25] or in different cellular models [26]. In general, ChIP assays utilize specific PCR primers flanking a suspected binding site to determine whether the site is occupied. Still, recent studies suggest that these regions, at least for mammalian genomes, are more complex than previously imagined and therefore ChIP-on chip may miss some regulatory regions. ChIP-Seq is another approach, but like ChIP-on chip, it requires high quality antibodies to the transcription factor of interest. The latter has been a big handicap particularly in the HH/Gli field; the development of ChIP grade of Gli antibodies is still a matter of intense research. To improve the number of target genes associated with the Shh/Gli pathway, we performed a yeast-based genomic screening assay combined with *in silico *analysis in order to characterize novel Gli interacting sequences. Yeast-based method has been successfully applied to identify transcription factor target genes in [9].

### Identifying new Hh targets using a yeast-based method

Our strategy shows that sequences obtained by the yeast based method possess potential binding sites for Gli transcription factor whose consensus matches the 9 bp core previously described. This method has the advantage that it allows transcription binding site identification for GBS that potentially cannot be found using other strategies. Of note, we found fragments that contain putative GBS sequences slightly different from what has been previously reported as consensus sequences (Figure 1B). Variations of these GBS lead us to examine whether a correlation existed between these changes and DNA binding domains for Gli family members. Zinc fingers 4 and 5, responsible for binding to the consensus sequence [27], are highly conserved specifically in the residues involved in contact with bases or phosphates. Lack of correlation between changes in individual consensus GBS sequences and DNA binding domains lead us to propose that these motifs might correspond to non-consensus binding sites for Gli1 with different affinities for the transcription factor. This hypothesis has been partially addressed in other works. In [25] for example, mutations of consensus GBS has been useful to predict potential targets for Gli family in mammalian enhancers, based in the effect of these mutations on activation of a luciferase reporter. These data indicate that variations from consensus GBS can affect gene expression levels, as also described in [6]. In [28], an essential binding site from *ptc1 *promoter, with a single substitution from the consensus GBS sequence, was mutated to investigate the effect of change bases in the expression of a reporter gene, resulting in different non-consensus and low affinity Gli binding sites that could lead to transcriptional activation.

Several models have been proposed for Hh activation of target genes. Gli proteins can bind to regulatory regions and directly either activate or repress transcription. In *Drosophila *[29] a differential affinity model seems to represent Hh/Ci signaling in the wing imaginal disc. It remains to be seen whether Hh signaling in vertebrates follows a similar mechanism. We cannot rule out a role of Gli repressors (Gli3 in particular) in the physiological regulation of the new Gli target genes identified in this paper. The fact that we see increased expression of *ptc1*, however, leads us to associate this regulation preferentially to Gli activator function. It remains to be defined whether Gli repressor assays yield the same targets as Gli activators.

A very interesting result of our work is the fact that we were able to mate yeast zebra fish or mouse Gli constructs to either homologous or heterologous libraries. Indeed, this reveals potential conservation of the interaction between the Gli transcription factors and their putative GBS among vertebrates. Multi-species conservation of the identified sequences was also addressed. The majority of the assayed genes showed low percentage of mice-rat-human similarity or murine only conservation. Some genes like *neo1 *showed a high percentage of conservation in mice-rat-human comparisons, while others like *sfrp2 *did not show any conservation, probably due to the partial presence of repeating elements. Some of these sequences, though, also show predicted repeating elements, which might account for the absence of conservation observed in the multiple alignments.

### Target gene expression

Further experiments using the Hh-responsive cell line C3H10T1/2 confirmed the results obtained from our screening, showing positive correlations of expression level loss to pharmacological loss of function of the Hh/Gli pathway. Interestingly, though, none of our chosen genes show an "early response" to cyc treatments, with reduced mRNA levels coming after 12 hours of treatment like *ptc1*. Instead they displayed a slower response to pathway inhibition, where expression is significantly reduced only after 24 hours of treatment. Similarly, 24 hours pur treatment showed a rather modest increase in target genes, such as *neo1 *and *c-myc*, in comparison to the well known described *ptc1 *up-regulation. This may suggest different levels of regulation, possibly due to the different genomic and regulatory contexts of the given promoters causing a different response dynamic than *ptc1*. Indeed, it has been reported that the expression of some Hh target genes depends on pathway activation (e.g. that of *gli1*), whereas other targets must be expressed prior to the induction of Gli activators, which then have their expression increased via positive feedback (e.g. *ptc1*). Thus, it will be interesting to discover possible similarities and differences in the transcription of different types of Hh target genes.

Our results clearly indicate the necessity for functionally testing individual genes and associated networks resulting from bioinformatics analysis. That some of the selected targets do not show increased mRNA levels when cells are treated with the pur agonist (like *sfrp2*) is likely due to additional signals regulating gene expression. Gain-of-function provides extra information on the mechanisms of Gli-mediated transcription of these target genes when compared to loss-of-function treatments. The fact that seven out of eight of the chosen genes show reduced mRNA after cyc treatment highlights the degree of accuracy that our screen assay provides for identifying target genes and corroborates our selection criteria.

A major finding of this study is the identification of several novel Shh target genes that we show to be important for embryonic development *in-vivo*. The use of zebrafish embryos as an *in-vivo *model to validate target genes has been previously reported [7], and our ISH provided a spatial-temporal resolution to changes observed at the transcript level in cell culture. Here we tested three candidate genes, selected based on their involvement in different processes during development. *sfrp2 *is involved in myoblast differentiation [30]. *c-myc *is involved in the cell cycle and critically regulates cell proliferation [31]. *neo1 *has been related to axon migration and more recently to neural progenitor proliferation in the CNS [32]. The Shh pathway has been classically implicated in both myogenesis and neurogenesis. Indeed, Shh loss of function in early development generates severe alterations in slow muscle development [33]. Patterning of the dorsal/ventral axis, specification of neuronal and oligodendroglial cell types, and proliferation of dorsal brain structures like the cerebellum, optic tectum and the neocortex also rely on Shh signaling [6,18]. In addition to the key functions of Shh in CNS development, is also implicated in oncogenesis. Shh signaling is deregulated in medulloblastomas (MB), embryonic tumors composed of primitive-appearing cells that arise in the cerebellum. Interestingly, a recent survey of the methylation status of tumor suppressors or oncogenes in human MBs has revealed among several epigenetic silencers sfrp family members [34].

All chosen candidate genes evaluated in the zebrafish embryo showed altered expression consistent with the *in-vitro *data obtained from C3H10T1/2. Their response *in-vivo *in the zebrafish embryo was a very context dependent response, with expression showing variation in some structures while remaining unaltered in others. Not all expression was ablated in *smu *mutants, however, so this might pertain to additional signals that could be regulating expression in a context-dependent manner in the zebrafish embryo. All three Gli family members have been shown to recognize the same GBS and share redundant functions. However, our data clearly show functional differences between Gli1 and Gli2 members, as evidenced by the zebrafish mutant analysis. Thus it is of great interest to further investigate the specific functions of each Gli transcription factor, sorting out their distinct and overlapping roles upon targets.

## Conclusions

Our study uncovered target genes previously linked to Shh signaling and, most importantly, previously unknown genes likely to play important roles during embryonic development. Several new candidates, including positive controls, whose modulation by Shh/Gli has been reported, were analyzed in the context of zebrafish development. These findings extend our knowledge of the Shh regulatory pathway function in development. Attaining a thorough understanding of Hh signaling is of vital importance for developing a mechanistic understanding of congenital abnormalities and diseases.

## Methods

### Yeast growth, complementation and colony sequencing

Yeast protocols (growth and mating) were performed as described in [9]. Plasmids used for yeast libraries are the same as in [9]. Sequencing was performed as described in [9].

### MEME & MAST analysis

Intronic and upstream regions were obtained either through the ENSEMBL database or the UCSC genome browser database. Motif searches were performed using standalone MEME suite software available at http://meme.sdsc.edu/meme/cgi-bin/meme.cgi. MEME was run with the following parameters: -dna -mod tcm -nmotifs 3 -minw 6 -maxw 14 -evt 1e100 -revcomp -time 7200 -maxsize 60000 -nostatus -maxiter 20. MAST was running using -remcorr and -norc parameters.

### Clustal alignment

Sequence from Gli1, Gli2 and Gli3 proteins were obtained from the ENSEMBL database. Alignment was performed with the Bioedit editor [9], with default options for Clustal. For Gene Ontology analysis, we used DAVID at http://david.abcc.ncifcrf.gov/home.jsp and AmiGO at http://amigo.geneontology.org/.

### C3H10T1/2 cell culture and treatment

The cell line was obtained from ATCC. Cells were grown in Dulbecco's Modified Eagle Media (DMEM) supplemented with 10% Fetal Bovine Serum (FBS). Cells were passaged at 80% confluence and passages 2 through 9 were used. Gain and loss of function experiments were performed according to [35] Briefly, considering that these cells are highly contact inhibited we performed experiments once the cell projections are touching, but before full confluence (aprox. 60% confluence). Cells were not serum deprived before treatments; cyc was added in presence of 10% FBS, whereas pur was given in 0.5% FBS. Cells were treated for 24 hrs in presence of 0.5% FBS with either 1.25 μg/mL of the protein synthesis inhibitor CHX alone or in combination with the Hh agonist.

### Quantitative real-time PCR (qPCR)

RNA was extracted using TRI Reagent (Ambion) according to the manufacturer's recommendations. RNA integrity and purity were electrophoretically verified by ethidium bromide staining and by OD260/OD280 nm absorption ratio. RNAs were then treated with Turbo DNA-free kit (Ambion) and reverse transcribed with Oligo-dT and Superscript II (Invitrogen). Transcripts of *Bacillus subtilis dap *gene were added to the total RNA of each sample (1:2,000) as spike-in controls to monitor the efficiency of reverse transcription. To generate the spike-in control RNA, the plasmid containing *B. subtilis *dap gene was purified from strain ATCC 87486. Linear template DNA was generated by digesting the plasmid with restriction enzyme NotI and RNA was produced by *in-vitro *transcription using T3 RNA polymerase. The specific forward and reverse oligonucleotide primers for target genes and for reference gene (Additional file 4) were designed using Primer Premier 5.0 software (Premier Biosoft International), based on GeneBank database sequences. Quantitative RT-PCR (qPCR) reactions were performed in a LightCycler system (Roche) using SYBR Green to monitor cDNA amplification. The amplification reaction contained: 1 μL of LightCyclerTM DNA Master SYBR^® ^Green I, 25 mM MgCl2, 10 μM of forward and reverse primers and 100 ng of cDNA in a total volume of 10 μL. The following standard thermal profile was used: denaturation at 95°C for 10 min, followed by 35 three-step cycles of template denaturation at 95°C with a 5 s hold, primer annealing at 58-64°C for 10 s and extension at 72°C for 10 s. Data were analyzed using the LightCycler Software (v3, Roche). The reaction efficiency was determined for each PCR reaction with LinRegPCR v7.5 [36]. Three technical replicates were done for each combination of cDNA and primer pair, and the quality of the PCR reactions was checked through analysis of the dissociation and amplification curves. The products were resolved by 2% agarose gel electrophoresis to confirm the DNA fragments of expected size. Transcript levels of target genes within a cDNA were normalized to the respective transcript level of *gapdh *as described in [37]. To test whether *gadph *behaves as a housekeeping gene in the analyzed samples, transcript levels of *gadph *and *dap *were measured by qPCR in each cDNA sample and the ratios of control transcript to the endogenous transcript *gadph *were calculated. The results indicated that the abundance of *gadph *mRNA remains stable between samples (data not shown). qPCR reactions were performed on material from at least two independent biological experiments. To assess differences among treatments, we applied a one-way ANOVA and a posteriori LSD Fisher test. A p-value < 0.05 was considered statistically significant. Data from the CHX experiment were processed as described above, except that results were expressed as a ratio of relative transcript levels between control and treated cells.

### Zebrafish embryos

Wild-type and mutant zebrafish embryos were maintained at 28°C as described in the Zebrafish Book [38], and staged accordingly [39]. Mutant lines used were smooth-muscle omitted (*smuhi1640*; [40,41], a loss-of-function smoothened allele, detour (dtrts269; [41]), a loss-of-function gli1 allele and you-too (*yot*; [42]), a negative dominant allele for gli2. Mutants were maintained as heterozygotes and heterozygous adults were crossed to produce homozygous mutant offspring. Homozygous mutant individuals were identified by morphological criteria (*dtr *and *yot*). Developmental time points are expressed as hours post-fertilization (hpf).

All animal procedures were in accordance with the Chilean legislation and were approved by Institutional Animal Care and Use Committees.

### RNA in situ hybridization

In situ hybridization was performed essentially as previously described [43]. Partial neogenin1 (neo1) cDNA was isolated by PCR and a digoxigenin-labelled anti-sense neo1 probe was synthesized from the cDNA clone. The clone was linearized with ApaI and transcribed with T7 polymerase (Roche, Hertfordshire, UK). c-myc cDNA clone was a kind donation from Dr. Nora Calcaterra. Plasmid was digested with EcoRI then anti-sense probe generated using T7 polymerase. sfrp2 clone, a donation from Dr. Corinne Houart, linearized with EcoRI and anti-sense probe transcribed using T7 polymerase.

### Pharmacological treatments

Zebrafish embryos were treated with 10 μM cyc (Infinity Pharmaceuticals, Inc., Cambridge, MA) by adding 1 μl of a 10 mM stock solution (in 95% EtOH) to 1 ml of E3 medium at defined time points. Control embryos were treated simultaneously with an equal volume (1 μl) of 95% EtOH (cyc carrier). For pur (Calbiochem, CA) treatments embryos were grown in E3 medium with 10 μM of the Hh agonist at indicated time points and control embryos were treated with 1% DMSO in E3 medium. Treatments were carried out in 6-well plates in 2 mL E3 medium (30 embryos/well) at 28.5°C. Embryos were fixed with 4% paraformaldehyde, dehydrated in MeOH and processed for in situ hybridization and/or antibody labelling. Effectiveness of cyc was verified by decreased *ptc1 *expression of treated embryos while pur showed to be specific to Hh signaling, as indicated by increased *ptc1 *expression (shown in Additional File 5).

### Imaging

Photographs were either taken with a Leica DFC 300 FX camera using a Leica MZ12 dissecting microscope. Images were processed with Photoshop 7.0 for Macintosh and Image J (NIH).

## Abbreviations

Hh: Hedgehog; Shh: Sonic Hedgehog; TC: Optic tectum; CNS: Central Nervous System; Ptc1: Patched1; Smo: Smoothened; WT: Wild type; hpf: hours post fertilization; *smu*: slow-muscle-omitted; *dtr*: detour; *yot*: you too; cyc: cyclopamine; qPCR: quantitative Real Time PCR.

## Authors' contributions

LAM, CC **and **VP **designed the experiments and drafted the manuscript**. LAM performed the yeast based screening. CHQ, VC and LAM **performed the **expression experiments in cell culture, **data analyses**, **and revised the manuscript**. CC, MO and LAM performed the zebrafish expression experiments. SB designed the yeast-based screenings and revised the manuscript **All authors have read and approved the final manuscript**.

## Supplementary Material

Additional file 1 **Table listing all binding sites found in Shh/Gli yeast assay through our screening strategy**. Total mouse GBS positive sequences mapped to specific genes based on shortest distance to the transcriptional start sites. Click here for file

Additional file 2 **GBS interspecies conservation**. *in silico *analysis of GBS found in the promoter of selected vertebrate genes. Click here for file

Additional file 3 **Direct regulation of novel Shh/Gli target genes in the CH310T1/2 murine cell line**. CHX 24 hours treatment confirm direct regulation for canonical Shh regulated genes. Click here for file

Additional file 4 **Table listing all primers used in this study**. Primers designed for control and novel Shh/Gli target gene quantitative PCR assays. Click here for file

Additional file 5 **Analysis of *in-vivo *drug response based on *ptc1 *expression**. Control of zebrafish long-term pharmacological Hedgehog gain and loss of function treatment. *ptc1 *readout-gene expression was verified by *in situ hybridization*. Click here for file
