# Supplementary material for: Yeast-based assay identifies novel Shh/Gli target genes in vertebrate development
Source: BMC Genomics. 2012 Jan 3;13:2. doi: 10.1186/1471-2164-13-2 (PMC3285088; doi:10.1186/1471-2164-13-2)
Supplement: Additional file 2 — GBS interspecies conservation. in silico analysis of GBS found in the promoter of selected vertebrate genes. [file 1471-2164-13-2-S2.PDF]

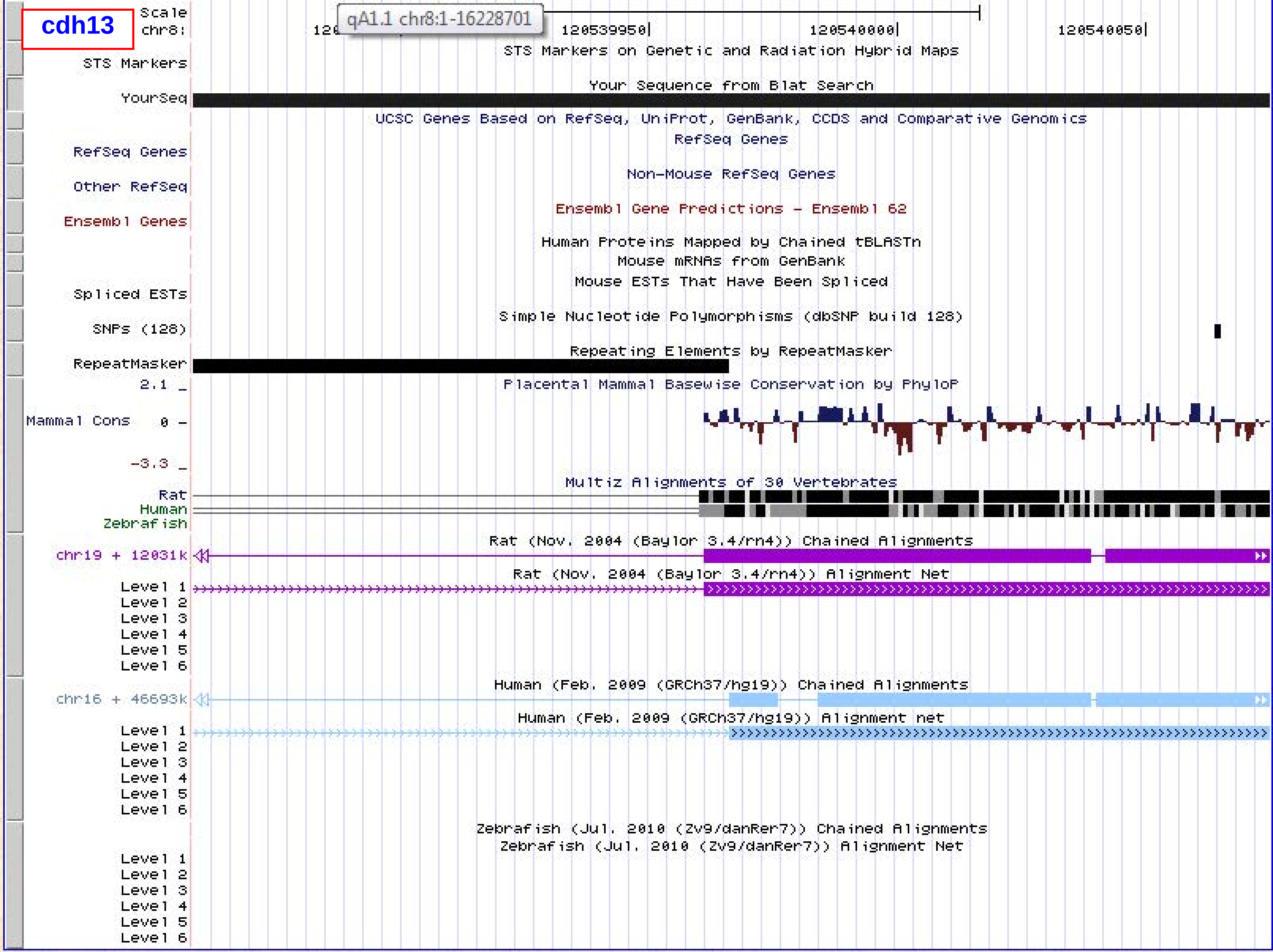
cdh13
Scale
chr8:
qA1.1 chr8:1-16228701
120539950
120540000
120540050
STS Markers on Genetic and Radiation Hybrid Maps
STS Markers
Your Sequence from Blat Search
YourSeq
UCSC Genes Based on RefSeq, UniProt, GenBank, CCDS and Comparative Genomics
RefSeq Genes
RefSeq Genes
Non-Mouse RefSeq Genes
Other RefSeq
Ensembl Gene Predictions - Ensembl 62
Ensembl Genes
Human Proteins Mapped by Chained tBLASTn
Mouse mRNAs from GenBank
Mouse ESTs That Have Been Spliced
Spliced ESTs
Simple Nucleotide Polymorphisms (dbSNP build 128)
SNPs (128)
Repeating Elements by RepeatMasker
RepeatMasker
Placental Mammal Basewise Conservation by PhyloP
2.1
Mammal Cons
0
-3.3
Multiz Alignments of 30 Vertebrates
Rat
Human
Zebrafish
Rat (Nov. 2004 (Baylor 3.4/rn4)) Chained Alignments
chr19 + 12031k
Rat (Nov. 2004 (Baylor 3.4/rn4)) Alignment Net
Level 1
Level 2
Level 3
Level 4
Level 5
Level 6
Human (Feb. 2009 (GRCh37/hg19)) Chained Alignments
chr16 + 46693k
Human (Feb. 2009 (GRCh37/hg19)) Alignment net
Level 1
Level 2
Level 3
Level 4
Level 5
Level 6
Zebrafish (Jul. 2010 (Zv9/danRer7)) Chained Alignments
Zebrafish (Jul. 2010 (Zv9/danRer7)) Alignment Net
Level 1
Level 2
Level 3
Level 4
Level 5
Level 6

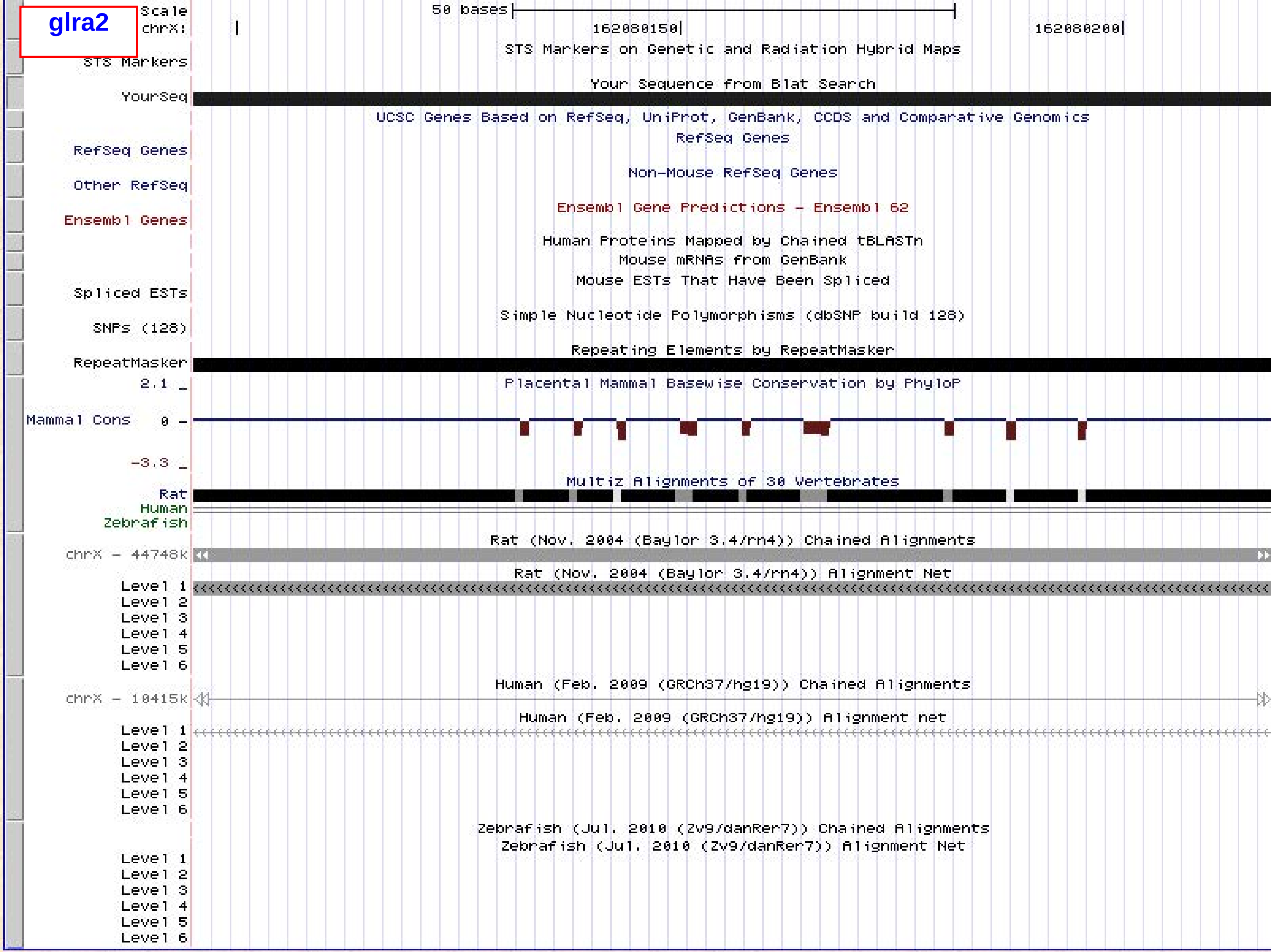
glra2
Scale
chrX:
50 bases
162080150
162080200
STS Markers on Genetic and Radiation Hybrid Maps
STS Markers
Your Sequence from Blat Search
YourSeq
UCSC Genes Based on RefSeq, UniProt, GenBank, CCDS and Comparative Genomics
RefSeq Genes
RefSeq Genes
Non-Mouse RefSeq Genes
Other RefSeq
Ensembl Gene Predictions - Ensembl 62
Ensembl Genes
Human Proteins Mapped by Chained tBLASTn
Mouse mRNAs from GenBank
Mouse ESTs That Have Been Spliced
Spliced ESTs
Simple Nucleotide Polymorphisms (dbSNP build 128)
SNPs (128)
Repeating Elements by RepeatMasker
RepeatMasker
2.1
Placental Mammal Basewise Conservation by PhyloP
Mammal Cons
0
-3.3
Multiz Alignments of 30 Vertebrates
Rat
Human
Zebrafish
Rat (Nov. 2004 (Baylor 3.4/rn4)) Chained Alignments
chrX - 44748k
Rat (Nov. 2004 (Baylor 3.4/rn4)) Alignment Net
Level 1
Level 2
Level 3
Level 4
Level 5
Level 6
Human (Feb. 2009 (GRCh37/hg19)) Chained Alignments
chrX - 10415k
Human (Feb. 2009 (GRCh37/hg19)) Alignment net
Level 1
Level 2
Level 3
Level 4
Level 5
Level 6
Zebrafish (Jul. 2010 (Zv9/danRer7)) Chained Alignments
Zebrafish (Jul. 2010 (Zv9/danRer7)) Alignment Net
Level 1
Level 2
Level 3
Level 4
Level 5
Level 6

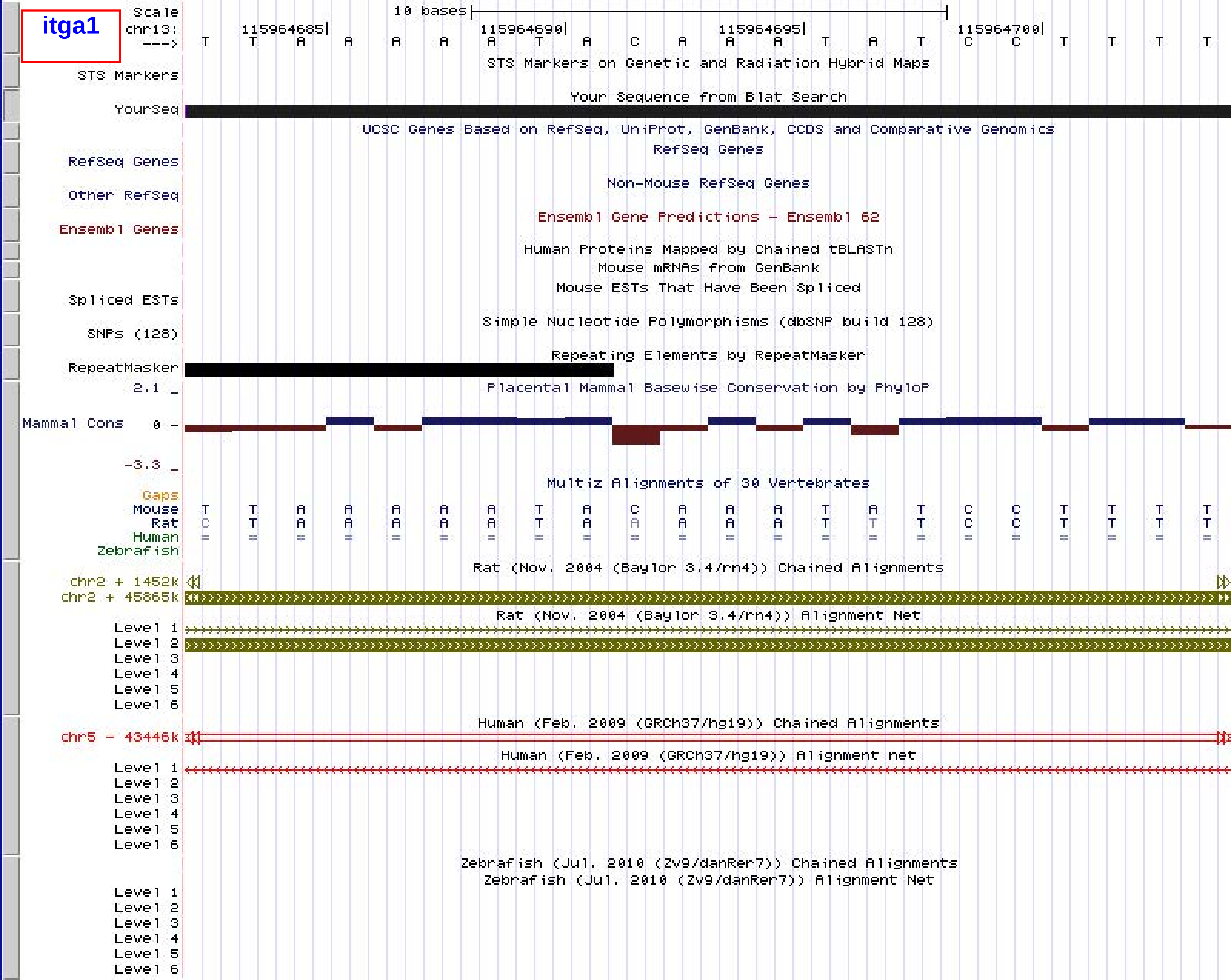
itga1
Scale
10 bases
chr13:
115964685
115964690
115964695
115964700
T T A A A A A T A C A A A T A T C C T T T T
STS Markers on Genetic and Radiation Hybrid Maps
STS Markers
Your Sequence from Blat Search
YourSeq
UCSC Genes Based on RefSeq, UniProt, GenBank, CCDS and Comparative Genomics
RefSeq Genes
RefSeq Genes
Non-Mouse RefSeq Genes
Other RefSeq
Ensembl Gene Predictions - Ensembl 62
Ensembl Genes
Human Proteins Mapped by Chained tBLASTn
Mouse mRNAs from GenBank
Mouse ESTs That Have Been Spliced
Spliced ESTs
Simple Nucleotide Polymorphisms (dbSNP build 128)
SNPs (128)
Repeating Elements by RepeatMasker
RepeatMasker
Placental Mammal Basewise Conservation by PhyloP
2.1
Mammal Cons
0
-3.3
Multiz Alignments of 30 Vertebrates
Gaps
Mouse
Rat
Human
Zebrafish
Rat (Nov. 2004 (Baylor 3.4/rn4)) Chained Alignments
chr2 + 1452k
chr2 + 45865k
Rat (Nov. 2004 (Baylor 3.4/rn4)) Alignment Net
Level 1
Level 2
Level 3
Level 4
Level 5
Level 6
Human (Feb. 2009 (GRCh37/hg19)) Chained Alignments
chr5 - 43446k
Human (Feb. 2009 (GRCh37/hg19)) Alignment net
Level 1
Level 2
Level 3
Level 4
Level 5
Level 6
Zebrafish (Jul. 2010 (Zv9/danRer7)) Chained Alignments
Zebrafish (Jul. 2010 (Zv9/danRer7)) Alignment Net
Level 1
Level 2
Level 3
Level 4
Level 5
Level 6

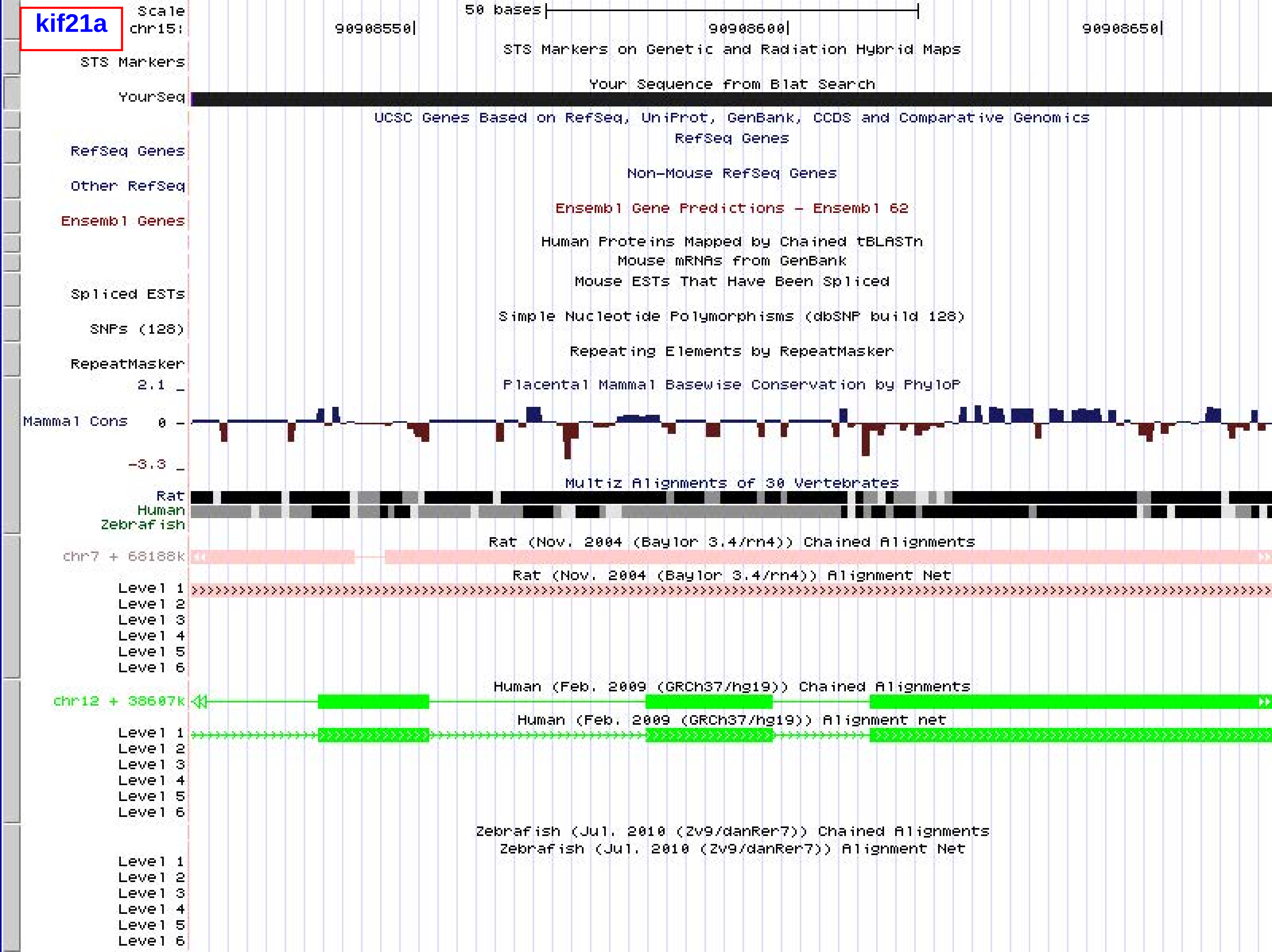
kif21a
Scale
chr15:
50 bases
90908550
90908600
90908650
STS Markers on Genetic and Radiation Hybrid Maps
STS Markers
Your Sequence from Blat Search
YourSeq
UCSC Genes Based on RefSeq, UniProt, GenBank, CCDS and Comparative Genomics
RefSeq Genes
RefSeq Genes
Non-Mouse RefSeq Genes
Other RefSeq
Ensembl Gene Predictions - Ensembl 62
Ensembl Genes
Human Proteins Mapped by Chained tBLASTn
Mouse mRNAs from GenBank
Mouse ESTs That Have Been Spliced
Spliced ESTs
Simple Nucleotide Polymorphisms (dbSNP build 128)
SNPs (128)
Repeating Elements by RepeatMasker
RepeatMasker
2.1
Placental Mammal Basewise Conservation by PhyloP
Mammal Cons
0
-3.3
Multiz Alignments of 30 Vertebrates
Rat
Human
Zebrafish
Rat (Nov. 2004 (Baylor 3.4/rn4)) Chained Alignments
chr7 + 68188k
Rat (Nov. 2004 (Baylor 3.4/rn4)) Alignment Net
Level 1
Level 2
Level 3
Level 4
Level 5
Level 6
Human (Feb. 2009 (GRCh37/hg19)) Chained Alignments
chr12 + 38607k
Human (Feb. 2009 (GRCh37/hg19)) Alignment net
Level 1
Level 2
Level 3
Level 4
Level 5
Level 6
Zebrafish (Jul. 2010 (Zv9/danRer7)) Chained Alignments
Zebrafish (Jul. 2010 (Zv9/danRer7)) Alignment Net
Level 1
Level 2
Level 3
Level 4
Level 5
Level 6

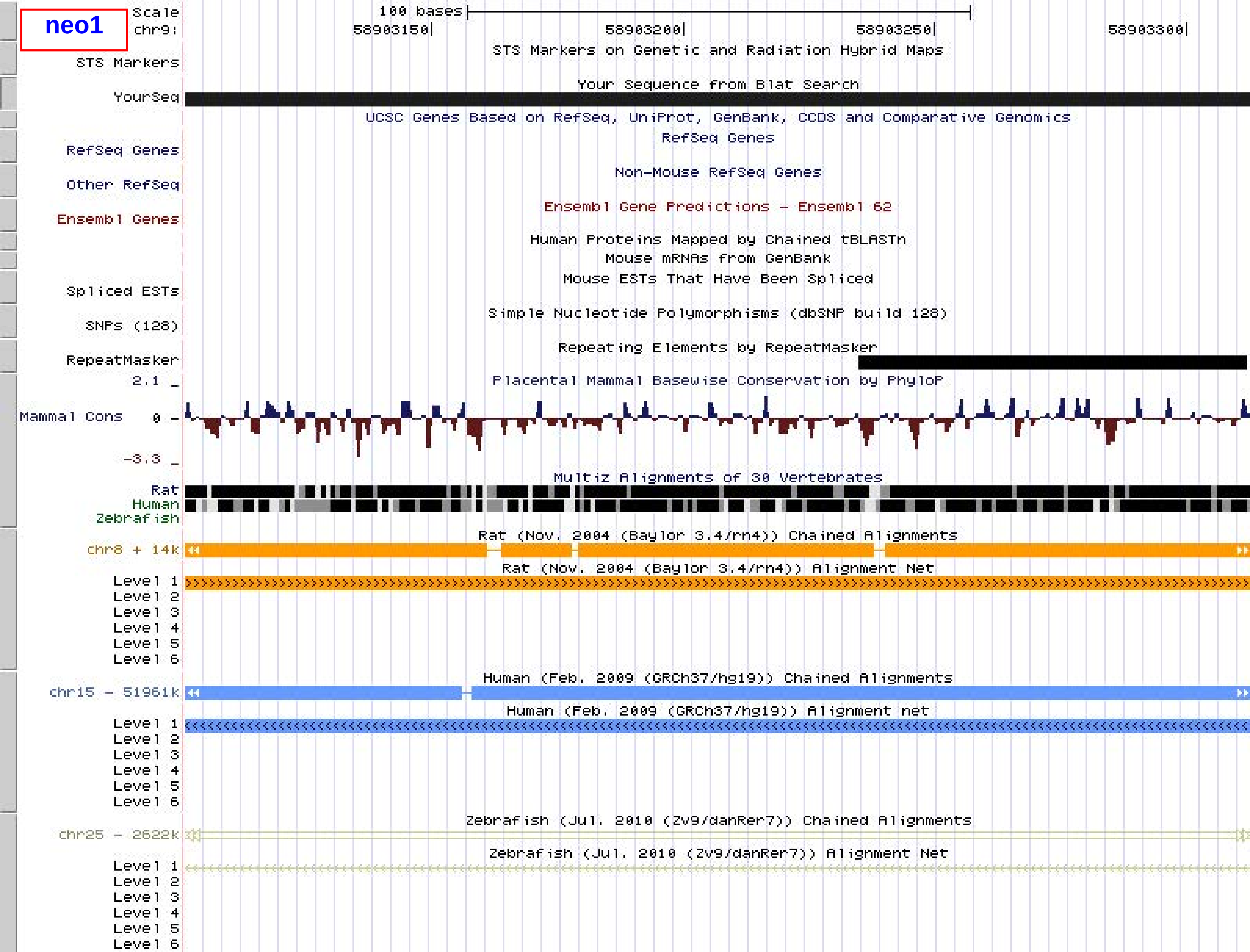
neo1
Scale
chr9:
100 bases
58903150
58903200
58903250
58903300
STS Markers on Genetic and Radiation Hybrid Maps
STS Markers
Your Sequence from Blat Search
YourSeq
UCSC Genes Based on RefSeq, UniProt, GenBank, CCDS and Comparative Genomics
RefSeq Genes
RefSeq Genes
Non-Mouse RefSeq Genes
Other RefSeq
Ensembl Gene Predictions - Ensembl 62
Ensembl Genes
Human Proteins Mapped by Chained tBLASTn
Mouse mRNAs from GenBank
Mouse ESTs That Have Been Spliced
Spliced ESTs
Simple Nucleotide Polymorphisms (dbSNP build 128)
SNPs (128)
Repeating Elements by RepeatMasker
RepeatMasker
2.1
Placental Mammal Basewise Conservation by PhyloP
Mammal Cons
0
-3.3
Multiz Alignments of 30 Vertebrates
Rat
Human
Zebrafish
Rat (Nov. 2004 (Baylor 3.4/rn4)) Chained Alignments
chr8 + 14k
Rat (Nov. 2004 (Baylor 3.4/rn4)) Alignment Net
Level 1
Level 2
Level 3
Level 4
Level 5
Level 6
Human (Feb. 2009 (GRCh37/hg19)) Chained Alignments
chr15 - 51961k
Human (Feb. 2009 (GRCh37/hg19)) Alignment net
Level 1
Level 2
Level 3
Level 4
Level 5
Level 6
Zebrafish (Jul. 2010 (Zv9/danRer7)) Chained Alignments
chr25 - 2622k
Zebrafish (Jul. 2010 (Zv9/danRer7)) Alignment Net
Level 1
Level 2
Level 3
Level 4
Level 5
Level 6

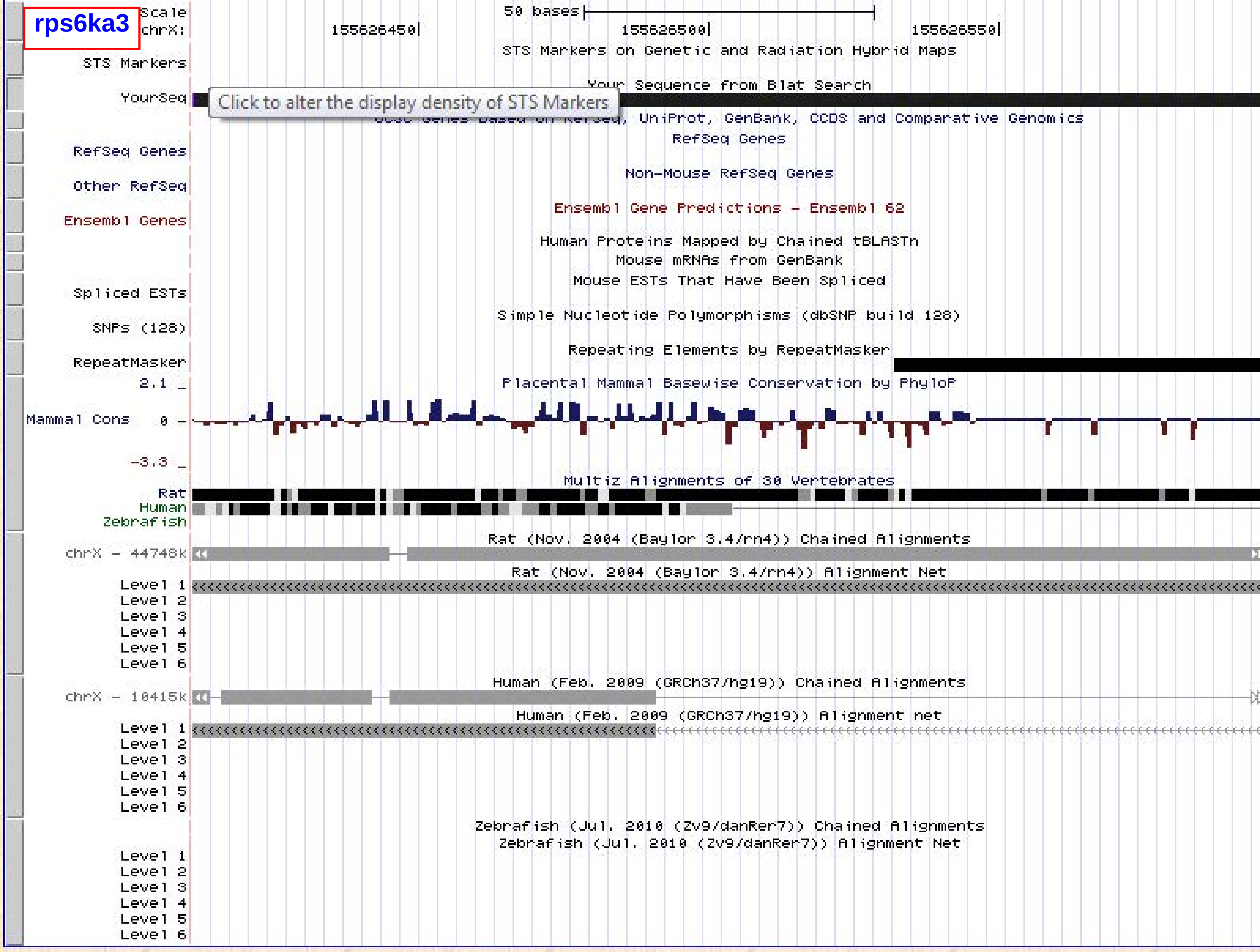

rps6ka3
Scale
chrX:
50 bases
155626450
155626500
155626550
STS Markers on Genetic and Radiation Hybrid Maps
STS Markers
Your Sequence from Blat Search
YourSeq
Click to alter the display density of STS Markers
UniProt, GenBank, CCDS and Comparative Genomics
RefSeq Genes
RefSeq Genes
Non-Mouse RefSeq Genes
Other RefSeq
Ensembl Gene Predictions - Ensembl 62
Ensembl Genes
Human Proteins Mapped by Chained tBLASTn
Mouse mRNAs from GenBank
Mouse ESTs That Have Been Spliced
Spliced ESTs
Simple Nucleotide Polymorphisms (dbSNP build 128)
SNPs (128)
Repeating Elements by RepeatMasker
RepeatMasker
2.1
Placental Mammal Basewise Conservation by PhyloP
Mammal Cons
0
-3.3
Multiz Alignments of 30 Vertebrates
Rat
Human
Zebrafish
Rat (Nov. 2004 (Baylor 3.4/rn4)) Chained Alignments
chrX - 44748k
Rat (Nov. 2004 (Baylor 3.4/rn4)) Alignment Net
Level 1
Level 2
Level 3
Level 4
Level 5
Level 6
Human (Feb. 2009 (GRCh37/hg19)) Chained Alignments
chrX - 10415k
Human (Feb. 2009 (GRCh37/hg19)) Alignment net
Level 1
Level 2
Level 3
Level 4
Level 5
Level 6
Zebrafish (Jul. 2010 (Zv9/danRer7)) Chained Alignments
Zebrafish (Jul. 2010 (Zv9/danRer7)) Alignment Net
Level 1
Level 2
Level 3
Level 4
Level 5
Level 6

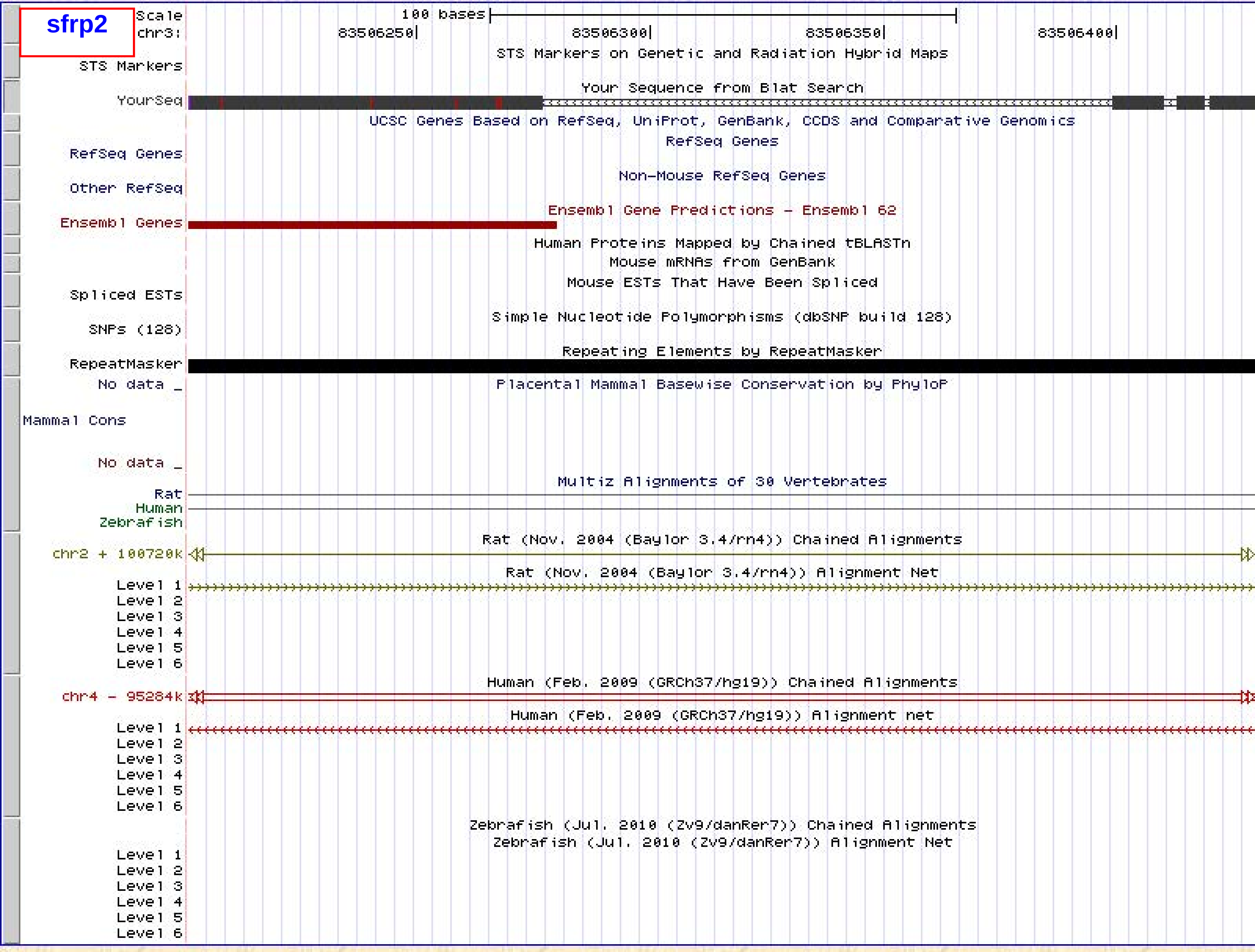
sfrp2
Scale
chr3:
100 bases
83506250
83506300
83506350
83506400
STS Markers on Genetic and Radiation Hybrid Maps
STS Markers
Your Sequence from Blat Search
YourSeq
UCSC Genes Based on RefSeq, UniProt, GenBank, CCDS and Comparative Genomics
RefSeq Genes
RefSeq Genes
Non-Mouse RefSeq Genes
Other RefSeq
Ensembl Gene Predictions - Ensembl 62
Ensembl Genes
Human Proteins Mapped by Chained tBLASTn
Mouse mRNAs from GenBank
Mouse ESTs That Have Been Spliced
Spliced ESTs
Simple Nucleotide Polymorphisms (dbSNP build 128)
SNPs (128)
Repeating Elements by RepeatMasker
RepeatMasker
No data _
Placental Mammal Basewise Conservation by PhyloP
Mammal Cons
No data _
Multiz Alignments of 30 Vertebrates
Rat
Human
Zebrafish
Rat (Nov. 2004 (Baylor 3.4/rn4)) Chained Alignments
chr2 + 100720k
Rat (Nov. 2004 (Baylor 3.4/rn4)) Alignment Net
Level 1
Level 2
Level 3
Level 4
Level 5
Level 6
Human (Feb. 2009 (GRCh37/hg19)) Chained Alignments
chr4 - 95284k
Human (Feb. 2009 (GRCh37/hg19)) Alignment net
Level 1
Level 2
Level 3
Level 4
Level 5
Level 6
Zebrafish (Jul. 2010 (Zv9/danRer7)) Chained Alignments
Zebrafish (Jul. 2010 (Zv9/danRer7)) Alignment Net
Level 1
Level 2
Level 3
Level 4
Level 5
Level 6
