# Supplementary material for: Yeast-based assay identifies novel Shh/Gli target genes in vertebrate development
Source: BMC Genomics. 2012 Jan 3;13:2. doi: 10.1186/1471-2164-13-2 (PMC3285088; doi:10.1186/1471-2164-13-2)
Supplement: Additional file 3 — Direct regulation of novel Shh/Gli target genes in the CH310T1/2 murine cell line. CHX 24 hours treatment confirm direct regulation for canonical Shh regulated genes. [file 1471-2164-13-2-S3.PDF]

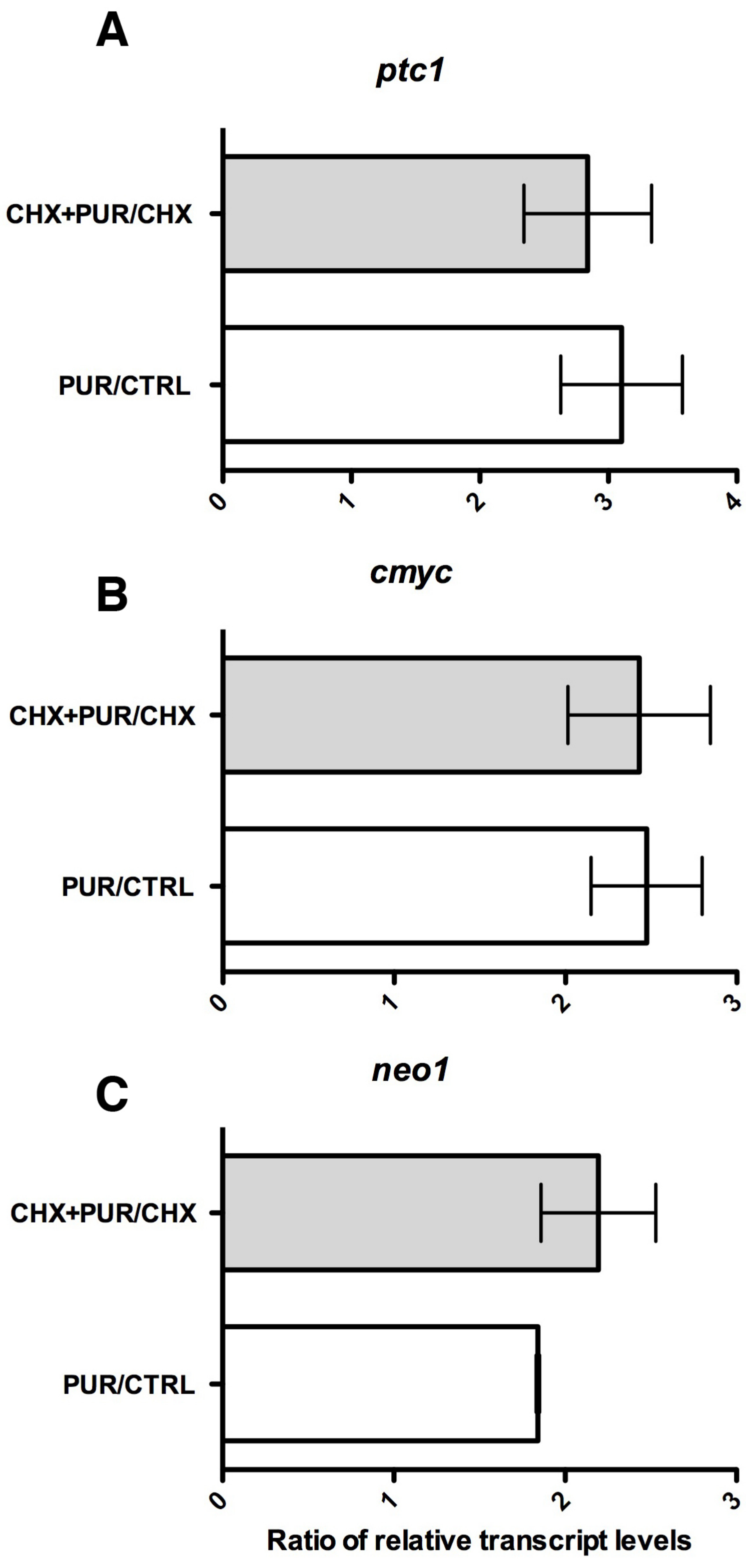
A
ptc1
CHX+PUR/CHX
PUR/CTRL
0
1
2
3
4
B
cmyc
CHX+PUR/CHX
PUR/CTRL
0
1
2
3
C
neo1
CHX+PUR/CHX
PUR/CTRL
0
1
2
3
Ratio of relative transcript levels

Additional File 3 Legend. **Pur antagonist upregulates target gene expression with or without the protein synthesis inhibitor cycloheximide.** (A) *ptc*, (B) *cmyc*, (C) *neo1*. Abreviations: CHX+PUR/CHX, ratio of cells treated with chx+pur, and cells treated with chx. Three independent experiments with s.d. in each condition are shown.
