# Supplementary material for: Yeast-based assay identifies novel Shh/Gli target genes in vertebrate development
Source: BMC Genomics. 2012 Jan 3;13:2. doi: 10.1186/1471-2164-13-2 (PMC3285088; doi:10.1186/1471-2164-13-2)
Supplement: Additional file 4 — Table listing all primers used in this study. Primers designed for control and novel Shh/Gli target gene quantitative PCR assays. [file 1471-2164-13-2-S4.DOCX]

Table 2. Primers used for RT-qPCR reactions, as described in Methods.

| **Gene name** | **GeneBank accession number** | **Forward primer (5`→3`)** | **Reverse primer (3`→5`)** | **Tm (ºC)** |
| --- | --- | --- | --- | --- |
| *c-myc* | NM_010849 | GGAAAACGACAAGAGGCGGAC | CTGGTCACGCAGGGCAAAA | 63 |
| *kif2* | NM_016705 | CGCTGATTACATTCCCAAGAG | GATGCTGAGAACGCAACTGA | 62 |
| *rps6ka3* | NM_148945 | ACAAGGGGTGGTTCACAGAG | GCATCATAACCTTGCCGTTT | 58 |
| *rps6ka1* | NM_009097 | CTGTGAGCCGCAGACGAACT | GGAGCCTGAGCCCAGAAGAG | 66 |
| *sfrp2* | NM_009144 | CGAAAGGGACCTGAAGAAA | CCAGATACGGAGCGTTGAT | 58 |
| *itga1* | NM_001033228 | TAAGGGCTAAAGGATGGATG | GGGCAGGTATGTACTGGAGTT | 64 |
| *Glra2* | NM_183427 | CCCTGGACCTTCTTGCCTAA | CCCTCGTGATTTCCCTCCC | 62 |
| *cdh13* | NM_019707 | TGGCAGAACTCGTGATTGTC | GGTGAGCCGGAACTTGGA | 63 |
| *ptc1* | NM_008957 | CTTCTCCTATCTTCTGACGGGT | AAAGAACTGCGGCAAGTTTTTG | 62 |
| *gapdh* | NM_008084 | ACCACAGTCCATGCCATCAC | TCCACCACCCTGTTGCTGTA | 58 |
