# Supplementary figures and images for: Yeast-based assay identifies novel Shh/Gli target genes in vertebrate development
Source: BMC Genomics. 2012 Jan 3;13:2. doi: 10.1186/1471-2164-13-2 (PMC3285088; doi:10.1186/1471-2164-13-2)

10hpf+cyc→48hpf

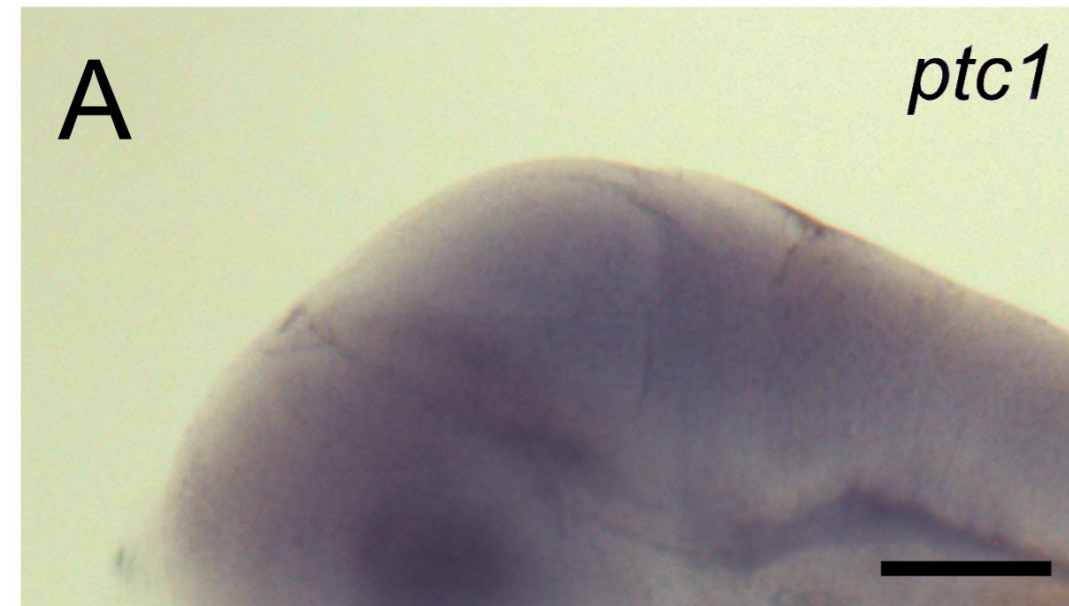

10hpf+EtOH→48hpf

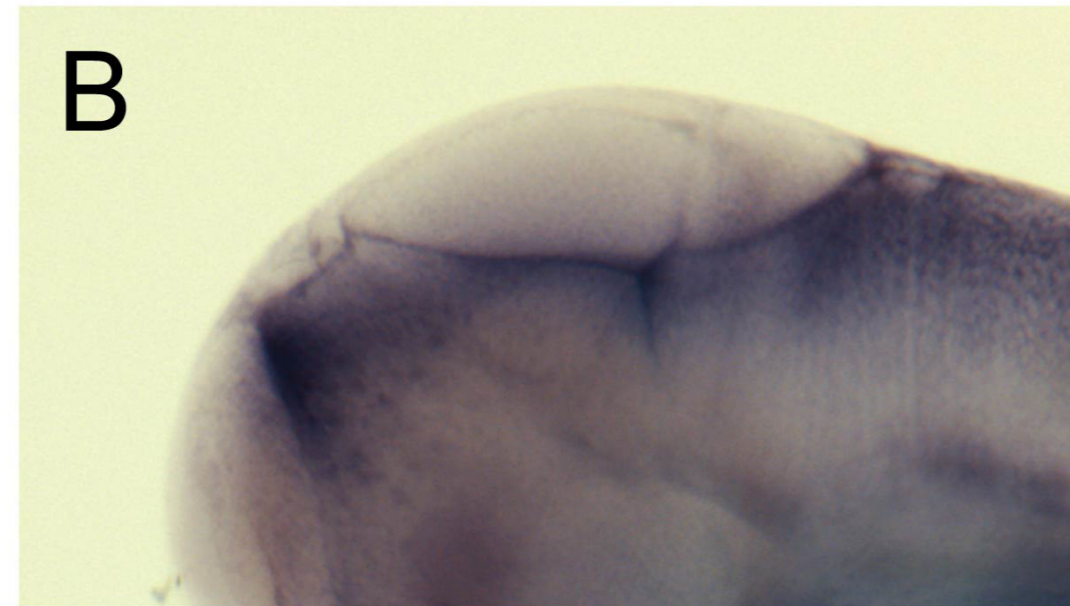

10hpf+pur→48hpf

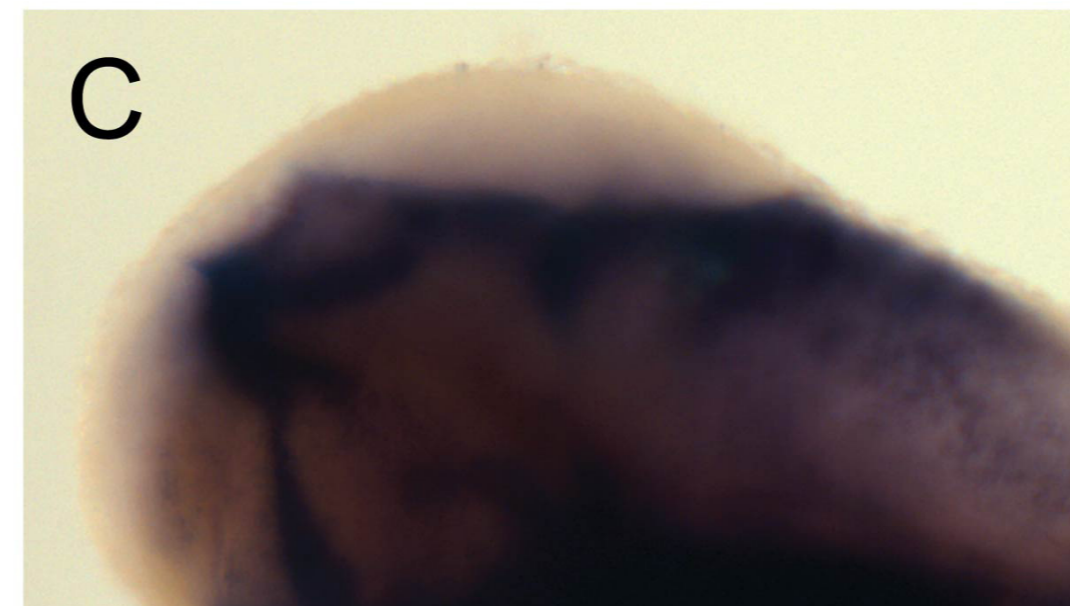

10hpf+DMSO→48hpf

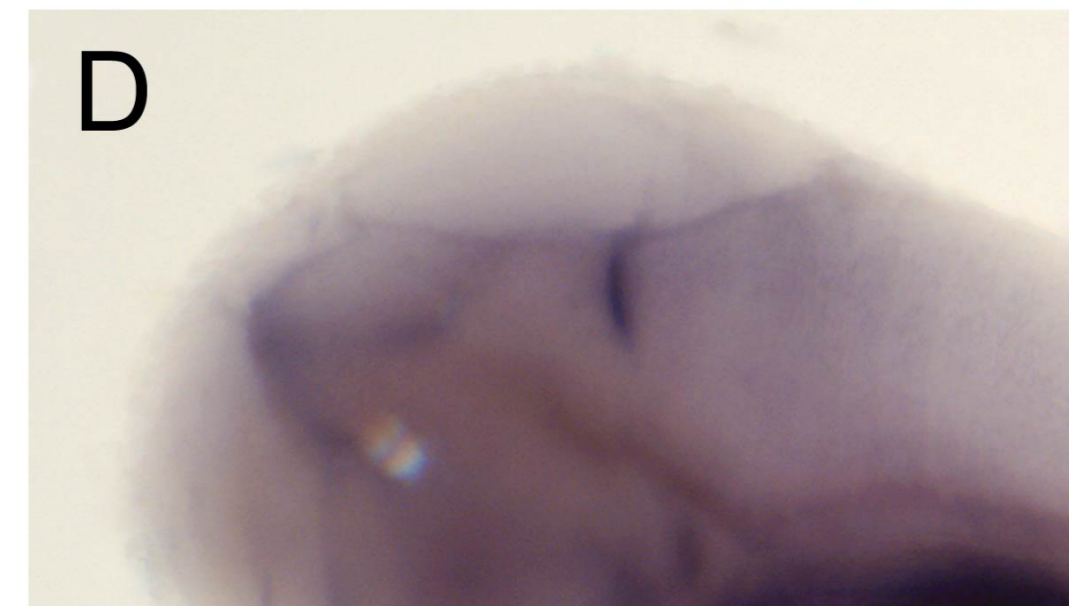

Supplement: Additional file 5 — Analysis of in-vivo drug response based on ptc1 expression. Control of zebrafish long-term pharmacological Hedgehog gain and loss of function treatment. ptc1 readout-gene expression was verified by in situ hybridization. [file 1471-2164-13-2-S5.PDF]
